# Exogenous Betaine Enhances the Protrusion Vigor of Rice Seeds under Heat Stress by Regulating Plant Hormone Signal Transduction and Its Interaction Network

**DOI:** 10.3390/antiox11091792

**Published:** 2022-09-11

**Authors:** Xu Mo, Jingya Qian, Peng Liu, Hongli Zeng, Guanghui Chen, Yue Wang

**Affiliations:** 1 Department of Agronomy, College of Agronomy, Hunan Agricultural University, Changsha 410128, China; 2 The Key Laboratory of Crop Germplasm Innovation and Resource Utilization of Hunan Province, Hunan Agricultural University, Changsha 410128, China

**Keywords:** rice, heat stress, betaine, seed germination, physiology, biochemistry

## Abstract

Rice is an important food crop. Rice seedlings are mainly composed of root, coleoptile, mesocotyl and euphylla. The elongation of coleoptile and mesocotyl promotes the emergence of rice seedlings. Therefore, analyzing the mechanism of coleoptile and mesocotyl elongation is important for the cultivation of rice varieties. Due to global warming, heat stress is threatening rice yields. Betaine plays an important role in plant resistance to heat stress; however, we lack research on its regulation mechanism of rice seed germination under heat stress. Therefore, we explored the effects of soaking seeds with betaine at different concentrations on rice seed germination under heat stress. According to the results, soaking seeds with 10 mM of betaine could effectively improve the seeds’ germination potential and rate under heat stress to promote the germination of rice seeds. To clarify the mitigation mechanism of betaine in heat stress, we measured the antioxidant enzyme activity, malondialdehyde content, soluble protein content and endogenous hormone content of seed protrusion under heat stress. We constructed the cDNA library for transcriptome sequencing. According to the results, 10 mM of betaine improved the activities of the superoxide dismutase, peroxidase and catalase of seed protrusion under heat stress to reduce the malondialdehyde content and increase the soluble protein content to alleviate the effect of heat stress on rice seed germination. The detection of the endogenous hormone content showed that soaking seeds with 10 mM of betaine increased the content of gibberellin and decreased the contents of auxin and abscisic acid of seed protrusion under heat stress. According to the transcriptome analysis, betaine can induce the expressions of key genes in the biosynthesis and metabolism of auxin, abscisic acid and gibberellins in the seed coleoptile and mesocotyl elongation stage, regulate the signal transduction of three hormones and promote the germination of rice seeds under heat stress. This study revealed, for the first time, the physiological and molecular regulation mechanism of betaine promotion of seed germination under heat stress.

## 1. Introduction

Rice (*Oryza sativa* L.) is one of the most important food crops, feeding more than half of the world′s population [1]. Rice is a thermophilic crop, and temperature is one of the most important factors affecting its growth and development. However, greenhouse gas emissions are warming the planet′s temperature because of a variety of human and natural forces. Researchers estimate that, by the end of the 21st century, the global average temperature will increase by 2–4 °C, and for every 1 °C increase in the minimum temperature in the growing season, the rice yield will be reduced by 10% [2,3]. According to estimates, the effects of global warming will cause a 41% reduction in the rice yield by the end of the 21st century [4].

The reduction in the rice yield caused by heat stress mainly affects the important period of rice grain formation. For example, heat stress at the booting stage of rice leads to the degeneration of spikelets, pollen abortion, a decline in pollen vitality and then to an increase in dry grains [5,6]. Heat stress during the tassel and filling stages can prevent pollination, increase the number of blighted grains in the spike and reduce the fruit set rate, which results in the reduced weight of a thousand seeds and, hence, a lower rice yield [7,8]. Therefore, in the future development of rice, high temperature will be one of the main factors limiting stable and high rice yields.

Temperature is one of the main factors affecting seed germination. The optimum temperature for the germination of rice seeds is 25 °C. When the temperature is 32 °C, the germination is fast and neat; however, when the germination period encounters heat stress of more than 35 °C, the germination of rice seeds is inhibited, which involves the accumulation of reactive oxygen species (ROS) and changes in plant hormone levels [9]. Heat stress decouples the enzymes and metabolic pathways of plants. Heat stress further induces and triggers an oxidative stress reaction by destroying the stability of the cell membrane through membrane lipid peroxidation and protein denaturation, which result in the accumulation of a large number of reactive oxygen species, such as singlet oxygen, superoxide radical, hydrogen peroxide (H_2_O_2_) and hydroxyl radical [10]. Efficient enzymatic and nonenzymatic antioxidant defense systems play an important role in scavenging and detoxifying ROS to reduce membrane lipid peroxidation and maintain ROS homeostasis and redox signaling [11]. Heat stress leads to a reduction in the activity of important members of the antioxidant enzyme system, such as superoxide dismutase (SOD), peroxidase (POD) and catalase (CAT), which can further lead to an imbalance in the ROS system, accelerating the accumulation of ROS and ultimately inhibiting seed germination [12,13].

In addition to the excessive accumulation of reactive oxygen species, the imbalance of endogenous hormone levels caused by heat stress is also an important reason for inhibiting seed germination. Abscisic acid (ABA) inhibits seed germination, and gibberellins (GAs) promote it. Abscisic acid and gibberellins act antagonistically with each other in the plant to regulate seed germination [14]. The inhibition of seed germination by heat stress is called the thermal inhibition of seed germination. Thermal inhibition inhibits the expression of GA biosynthesis genes and ABA inactivation genes; however, it promotes the expression of ABA synthesis genes [15]. The mitigation of heat stress by ABA is accomplished through synergistic effects with nitric oxide (NO) to increase the antioxidant enzyme activity, reduce the levels of H_2_O_2_ and thiobarbituric acid reactive substances (TBARS), and increase carbohydrates, adenosine triphosphate and heat-shock proteins in plants [16,17]. Auxin (IAA) is the second hormone after ABA that positively regulates seed dormancy [18]. In response to heat stress induction, the expression levels of the growth hormone synthesis genes *OsIAA13* and *OsIAA20* were increased, and the growth hormone response gene *SAUR* was also substantially induced to be expressed [19]. The elongation and growth of *Arabidopsis* hypocotyl under heat stress is related to the increase in the auxin content [20].

Betaine is a quaternary amine compound that is commonly found in bacteria, fungi, higher plants and animals [21]. It plays an extremely important role in plant resistance to heat stress. The heat tolerance of tomato lines transformed with the *codA* gene of the betaine synthesis gene was significantly higher than that of tomato lines without betaine [22]. During the germination of *Arabidopsis* seeds, transgenic plants with the *codA* gene increased the level of betaine, thereby enhancing the high-temperature tolerance of the seeds and promoting seed germination [23]. Endogenous betaine can enhance the high-temperature tolerance of plants. After spraying betaine on leaves, the photoinhibition caused by the heat stress of *Tagetes erecta* was reduced, and the CO_2_ assimilation rate, stomatal conductance and transpiration rate improved. Betaine also reduced the levels of hydrogen peroxide, superoxide, lipid peroxidation and cell death [24]. Spraying betaine on tobacco leaves significantly increased the activities of SOD and POD, increased the contents of chlorophyll and proline, and decreased the contents of malondialdehyde (MDA), alleviating heat stress and promoting the accumulation of tobacco biomass under the combined stress of high temperature and drought [25]. Spraying leaves with 10mM betaine improved the average total number of florets obtained in summer maize Jingnongke 728 under the different treatments of early sowing date, normal sowing date and late sowing date, increasing by 5.09%, 4.70% and 2.27%, respectively. The yield of summer maize Jingnongke 728 after spraying betaine increased by 3.05–12.81% [26]. Rice is a typical non-betaine-accumulating species [27].

At present, researchers conduct investigations into betaine to alleviate the stress damage suffered by rice by introducing the betaine enzyme system into rice or by exogenous application through transgenic technology. Transforming the *BADH* gene encoding betaine aldehyde dehydrogenase from barley into rice promoted the accumulation of betaine in rice and significantly improved the tolerance of rice to salt, cold and heat stress [28]. The exogenous application of betaine can also effectively improve the adaptability of rice. Spraying 50 mM or 100 mM concentrations of betaine on the leaves effectively enhanced the osmotic adjustment ability of rice and improved its adaptability to osmotic stresses, such as salt, drought and heat stress [29,30,31]. These reports mostly investigate the effect of betaine on rice-plant morphogenesis; however, we lack research on the seed germination stage before seedling morphogenesis, and we do not yet understand the molecular mechanism of betaine to alleviate stress damage. In order to study the mechanism of exogenous betaine during rice seed germination, we took XZX45 as the test material, soaked seeds with different concentrations of betaine and carried out seed germination tests at different temperatures. We further measured the antioxidant enzyme activity, MDA content, soluble protein content and endogenous hormone content under heat stress. After heat stress at 38 °C for 24 h, we constructed the cDNA libraries for transcriptome sequencing. We identified differentially expressed genes (DEGs) related to plant endogenous hormone synthesis and metabolism, plant hormone signal transduction and reactive oxygen species accumulation, and we verified the credibility of these differentially expressed genes by qRT-PCR. Finally, we clarified the physiological characteristics of soaking seeds in betaine solutions to alleviate the seed coleoptile and mesocotyl elongation stage under heat stress, and we revealed the molecular mechanism of the betaine promotion of seed germination by regulating plant hormone synthesis and metabolism, as well as signal transduction.

## 2. Materials and Methods

### 2.1. Test Materials

The tested material was XZX45, which is a rice variety that is mainly planted in rice-growing areas in the middle and lower reaches of the Yangtze River in China. The high-quality variety Zhouyou 903 was used as the female parent to prepare an inter-variety hybrid combination with Zhefu 504, which had a short growth period and strong resistance. The offspring was named XZX45 after purification. The entire growth period of XZX45 is 106 days, which is characterized by strong lodging resistance and excellent rice quality. In China’s provincial regional test of varieties, the average yield was 7573.5 kg ha^−1^. It is also mainly used as the national grain reserve. All test seeds were harvested in 2021.

### 2.2. Experimental Design

We carried out seed germination in a germination box (length, width and height of 12 cm, 12 cm and 5 cm, respectively). According to the previous research results of this experiment, the betaine seed-soaking solution was set at 0 mM, 0.1 mM, 1 mM and 10 mM. We selected seeds with full grains, soaked them and disinfected them with 5% sodium hypochlorite for 30 min, and we then rinsed them 5 times with sterile distilled water. After soaking the seeds with betaine solutions of 0 mM, 0.1 mM, 1 mM and 10 mM concentrations for 24 h, we placed them in germination boxes with sterilized germination paper, placing 100 seeds in each germination box. We added 7 mL of sterile distilled water to each germination box for seed germination, and we repeated each treatment 20 times. We conducted the germination test in a light incubator. We set the temperature for the heat stress treatment at 26 °C, 32 °C, 35 °C and 38 °C in 16 h light/8 h dark, and the humidity was 70%. The germination of seeds is subject to the condition that the bud length is equal to half of the seed length, and the root length is equal to the seed length. We recorded the germination every 24 h after sowing, and we counted the final germination rate on the seventh day of germination. We referred to the method of Yu et al. (2022) to determine the germination potential and rate [32]. The germination potential and germination rate were investigated on days 3 and 7 after initiation, respectively. The germination potential was calculated as germination potential (%) = (number of seeds germinated on day 3/total number of experimental seeds) × 100. The germination rate was calculated as germination rate (%) = (number of seeds germinated on day 7/total number of experimental seeds) × 100. After 24 h of the high-temperature treatments of the seeds, we measured and sampled the antioxidant enzyme activity, MDA content, soluble protein content, endogenous hormone content, transcriptome determination and related gene expressions. We quickly put the samples into a 2 mL centrifuge tube with RNase inactivation. After quick freezing with liquid nitrogen, we placed them in a refrigerator at −80 °C for storage.

### 2.3. Determination of Physiological Indexes

We determined the activity of superoxide dismutase (SOD) according to the method of Dhindsa et al. [33]. We referred to the method of Kar et al. [34] to determine the activity of peroxidase (POD). We used the UV absorption method [35] to determine the activity of catalase (CAT). We determined the content of malondialdehyde (MDA) by using the method of Li et al. [36]. We determined the content of soluble protein by the Coomassie brilliant blue G–250 method [37]. We repeated each measurement 5 times.

### 2.4. Determination of Endogenous Hormones

We accurately weighed 50 mg of the sample that we had ground and crushed in liquid nitrogen. We conducted the extraction and quantification of the endogenous IAA, ABA and GAs according to the operation guidelines of Wuhan Metwell Biotechnology Co., Ltd., China. We performed data collection using ultra-performance liquid chromatography (UPLC) (the Shim–pack UFLCSHIMADZU CBM30A system, Kyoto, Japan) and tandem mass spectrometry (MS) (Applied Biosystems, Waltham, MA, USA). Wuhan Metwell Biotechnology Co., Ltd., Wuhan, China (http://www.metware.cn/) (accessed on 17 November 2021) analyzed the data. We performed three biological replicates for each treatment.

### 2.5. RNA-Seq Analysis

The samples used for RNA-seq were seed embryos soaked in 0 mM and 10 mM of betaine, heat-stressed at 38 °C for 24 h. We labeled the treatments BT and HT+BT, respectively, and we performed three biological replicates of each treatment. We extracted the total RNA from young ear tissue with Trizol (Invitrogen, Waltham, MA, USA) reagent. Wuhan Huada Gene Technology Co., Ltd. completed the RNA-seq-library construction and sequencing. The screening conditions of differential genes were a Q value ≤ 0.05 and a fold change (|log_2_ ratio|) >1.5. We used the FPKM values to analyze the differential gene expression levels, and we used gene ontology (GO) enrichment analysis (agriGO v2.0: a GO analysis toolkit for the agricultural community, 2017 update) to analyze the biological processes of the differential gene participation. Kyoto Encyclopedia of Genes and Genomes (KEGG) pathway functional enrichment analyses were performed via the KEGG Database (https://www.genome.jp/kegg (accessed on 14 August 2022)) [38]. We used the phyper function in the R software for enrichment analysis and to calculate the *p*-value, and then FDR corrected the *p*-value, considering a Q value ≤ 0.05 as significant enrichment.

### 2.6. Quantitative Real-Time PCR

A total of nine genes were differentially expressed and subjected to qRT-PCR verification. Total RNA was extracted using the RC411-01 RT-PCR reagent (Vazyme Biotech, Nanjing, China). The cDNA was synthesized according to the instructions of the M-MLV reverse transcriptase Kit (28025013) (Thermo Fisher Scientific, Waltham, MA, USA). The qRT-PCR was FastStart Universal SYBR Green Master (Rox) superMIX reaction kit (4913850001) (Roche, Mannheim, Germany). The *Actin* gene was used as an internal reference. The primer pairs were designed using Primer Premier 5.0 software. The specific primers for genes involved in the hormone signaling pathway are listed in Table 1. Each biological sample was tested in triplicate, and the standard deviation (SD) values of the means were calculated using standard statistical methods. The expression of genes was analyzed using the DDCt data analysis method, and gene relative expression was calculated using the 2^−ΔΔCt^ method.

### 2.7. Data Processing and Analysis

We used Excel 2017 software for the data statistics, and the data drawing application Graphpad Prism 8.4.3. We used SPSS 21.0 (IBMCorp., Armonk, NY, USA) software to analyze the significant differences in the data based on the one-way analysis of variance (ANOVA) and Duncan methods, and the significant difference level was *p* < 0.05.

## 3. Results

### 3.1. Betaine Soaking Treatment Can Promote the Germination of Rice Seeds under Heat Stress

**Figure 1:**
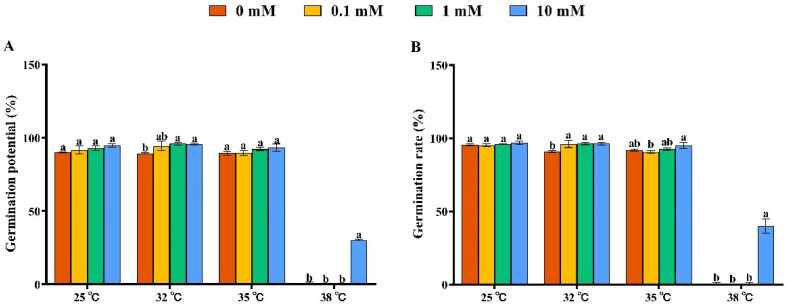
Effects of soaking seeds with different concentrations of betaine at 25 °C, 32 °C, 35 °C and 38 °C on germination characteristics of rice seeds. The difference in the average value of each letter indicates that there is a significant difference in the parameters (*p* < 0.05): (**A**) germination potential; (**B**) germination rate.

Germination potential and germination rate are two of the main indicators of seed quality. When germination rates are identical, seeds with high germination potential demonstrate greater vigor. Germination potential determines the regularity of seedling emergence [39]. Therefore, when the germination potential and germination rate of rice seeds are high, it indicates that the seeds emerge quickly and evenly. Meanwhile, the seedlings are robust. If rice seeds have low germination potential and a high germination rate, it indicates uneven seedling emergence and weaker seedlings. After soaking seeds in betaine at different concentrations, we conducted the germination test for rice seeds at different temperatures (Figure 1A,B). At 25 °C, soaking seeds with betaine at various concentrations had little effect on the germination rate and potential. At 32 °C, soaking seeds with betaine at all concentrations improved the germination rate and potential, which were 5.82–6.19% and 5.82–7.26% higher than the control (0 mM betaine), respectively. At 35 °C, soaking seeds with 0.1 mM, 1 mM and 10 mM of betaine had no significant effect on the germination potential and rate. At 38 °C, soaking seeds with 10 mM betaine significantly improved the germination potential and rate, which were 57.96 times and 43.77 times higher than the control (0 mM betaine), respectively.

### 3.2. Betaine Increased the Antioxidant Enzyme Activity and Soluble Protein Content of Rice Seeds under Heat Stress

**Figure 2:**
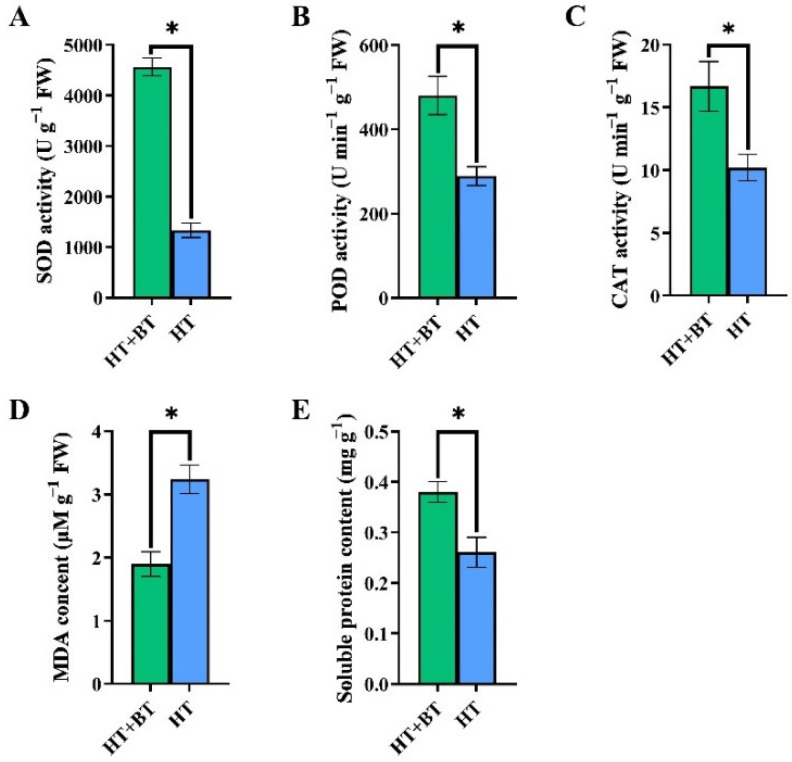
Effects of soaking seeds with 10 mM betaine on antioxidant enzyme system and soluble protein content of rice seeds under heat stress. Each parameter * indicates significant statistical difference, * (*p* < 0.05): (**A**) SOD activity; (**B**) POD activity; (**C**) CAT activity; (**D**) MDA content; (**E**) soluble protein content.

The seed germination activities of the 0 mM and 10 mM betaine soaking treatments were significantly different under heat stress for 24 h. By measuring the antioxidant enzyme activity, MDA content and soluble protein content, we found that the activities of SOD, POD and CAT in the HT + BT treatment were significantly higher than those in the HT treatment (Figure 2A–C) and were increased by 243.06%, 66.10% and 63.37%, respectively. The MDA content in the HT+BT treatment was significantly reduced by 70.69% compared with the HT treatment (Figure 2D), while its soluble protein content was significantly increased by 45.88%, compared with the HT treatment (Figure 2E).

### 3.3. Hormone Content during Seed Germination

**Figure 3:**
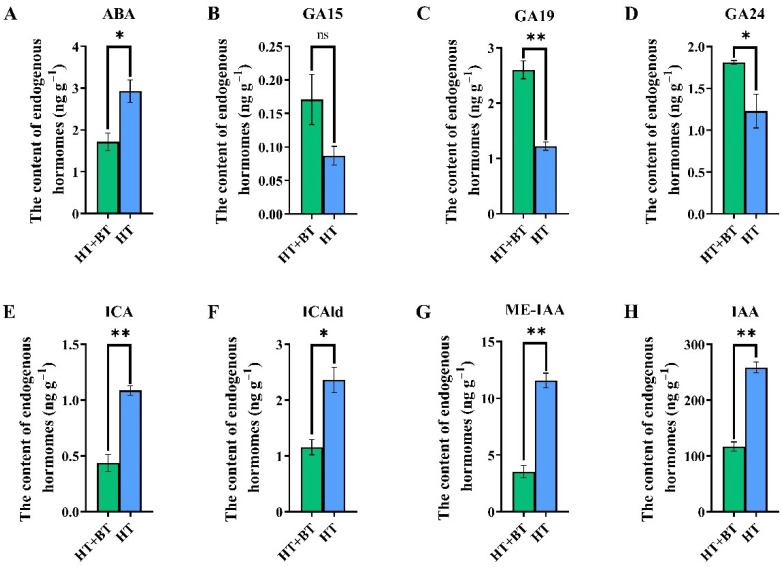
Comparison of hormone levels in HT- and HT + BT-treated seeds: (**A**) comparison of ABA content; (**B**) comparison of GA15 content; (**C**) comparison of GA19 content; (**D**) comparison of GA24 content; (**E**) comparison of ICA content; (**F**) comparison of ICAld content; (**G**) comparison of ME–IAA content; (**H**) comparison of IAA content. Each parameter * indicates significant statistical difference, * (*p* < 0.05), ** (*p* < 0.01).

Seed germination is regulated by endogenous plant hormones, so we measured the ABA, IAA and GA contents of the seed protrusion of both the HT- and HT + BT-treated seed endosperm breakthroughs. Under heat stress, the contents of ABA and IAA in the seeds were significantly higher than those of the betaine treatment, of which the ABA was 1.7 times that of the betaine treatment (Figure 3A), and the levels of indole-3-carboxylic acid (ICA), indole-3-carboxaldehyde (ICAld), methyl indole-3-acetic acid ester (ME–IAA) and IAA were 2.49 times, 2.04 times, 3.28 times and 2.21 times that of the betaine treatment, respectively (Figure 3E,H). The contents of various GAs were significantly inhibited under heat stress. Except for gibberellic acid 15 (GA15), the contents of gibberellic acid 19 (GA19) and gibberellic acid 24 (GA24) in the betaine treatment were significantly higher than those in the heat-stress treatment and were 2.13 times and 1.47 times higher than those in the HT treatment, respectively (Figure 3B–D).

### 3.4. Transcriptome Analysis of the Effect of Betaine Treatment on Rice Seed Germination under Heat Stress

During seed germination, we performed a transcriptomic analysis of the seed coleoptile and mesocotyl elongation stage after 24 h of immersion under heat stress at 38 °C. We performed three biological replicates of each treatment (Table 2). We present the sequencing data outputs and quality for the six samples in the table. Each sample produced an average of over 44 Mil. total raw reads. After quality control to remove low-quality reads, splice contamination and reads with high unknown base N content, we obtained an average of more than 6 Gb of total clean bases per sample, and all six samples had a Q30 of more than 85% with homogeneous base content.

### 3.5. Comparative Analysis of Differential Genes

**Figure 4:**
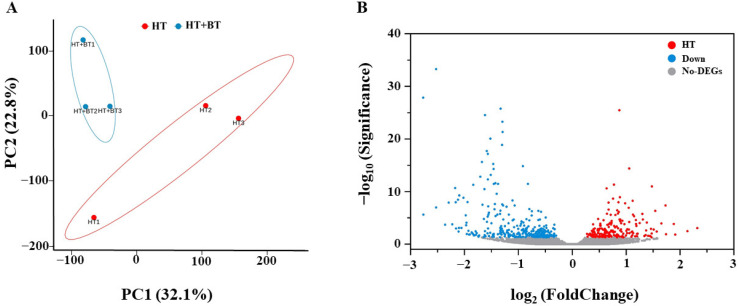
(**A**) HT and HT + BT treatments were the sources of gene expression variation under heat stress. We used principal component analysis to explore the impact on the variation. (**B**) Volcano plots of differentially expressed genes (DEGs) between HT and HT + BT; each point represents a gene. The upregulated genes are represented by red dots, the downregulated genes are represented by blue dots, and the genes with no significant differences are represented by gray dots. Under the corrected Q value, the DEG is considered significantly different.

We performed principal component analysis (PCA) on the gene expressions of the six samples to identify outliers and to discriminate between clusters of samples with high similarity. We found significant differences between the HT and HT+BT groups, with the two groups forming separate clusters. Moreover, the spatial distribution of the three replicates within the groups was more concentrated, which indicates that the results are reproducible (Figure 4A). In the differential gene volcano plots, we can see that the 10 mM betaine treatment induced the differential expressions of a total of 528 genes under heat stress, of which 332 genes were downregulated, and 196 genes were upregulated. This implies that betaine under heat stress promotes rice seed germination through the differential expressions of these 528 genes (Figure 4B).

### 3.6. GO Pathway Analysis

**Figure 5:**
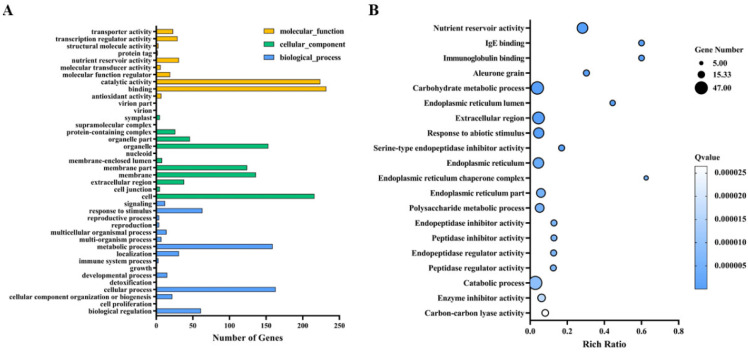
GO term comparison of differential genes with and without betaine soaking treatment under heat stress: (**A**) GO classification of differential genes; (**B**) bubble diagram of differential gene GO enrichment.

Functional annotation of transcriptome sequences is an important aspect of functional genomics. GO analysis is currently the main method of functional annotation. GO is a standard classification system of gene functions, which can comprehensively describe the properties of biological genes and gene products [40]. GO analysis includes the classification annotation of GO function and the significance enrichment analysis of GO function. The GO function classification annotation can obtain the gene list of the number statistics of genes with a certain GO function. The GO function significance enrichment analysis gives the GO items of genes that are significantly enriched compared with genomic background, which can obtain the biological functions of genes. The GO database is based on GO terms, which is a tree structure with redundancy. There are three root nodes in the GO database, which describe the molecular function, cellular component and biological process of genes. The GO term is a gene set, which is generally used to find functional changes caused by different genes [41,42]. For example, the molecular function of a gene may be catalytic activity. Its cellular components are localized to the cell membrane in the cell, and the biological process involved is protein trafficking. The three levels of GO are not related to each other, and there is no definition of the mutual relationship of genes. In order to visualize the data and obtain complete functional information, we performed a GO analysis to unify the gene attributes and classify differential genes into putative functional taxa. We divided the functional annotations into three broad categories: biological processes, cellular components and molecular functions. We annotated 528 significantly differentially expressed genes (DEGs) to 40 functional groups within the three broad functional categories (Figure 5A,B). A total of 16 were biological processes, 14 were cellular components and 10 were molecular functions. The majority of DEGs in biological processes were associated with cellular processes (29.06%) and metabolic processes (28.34%); the majority of DEGs in cellular components were associated with cells (34.29%), membranes (16.67%), membrane fractions (14.87%) and organelles (18.35%); the majority of DEGs in molecular functions were associated with binding (40.28%) and catalytic activity (38.89%).

### 3.7. KEGG Pathway Analysis

**Figure 6:**
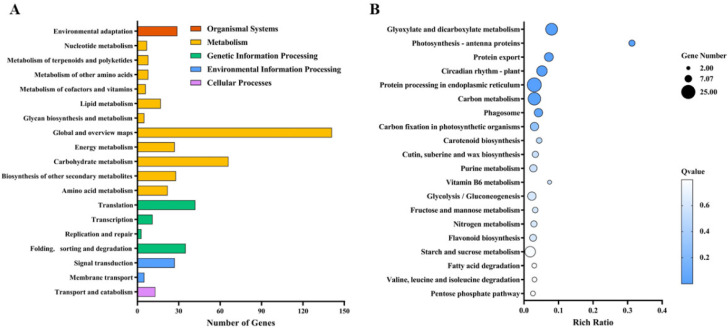
KEGG term comparison of differential genes with and without betaine soaking treatment under heat stress: (**A**) KEGG classification of differential genes; (**B**) bubble diagram of differential gene KEGG enrichment.

KEGG is a signaling pathway database with an extremely rich mapping of signaling pathways and of the interactions between the genes contained in the pathways [38]. KEGG analysis allows us to visualize the expressions of genes and their regulatory pathways. According to the results, 290 DEGs were enriched in 90 pathways (Figure 6A,B). Seed germination is regulated by factors such as ambient temperature and endogenous hormones. In the signal transduction pathway of environmental information, we found that many different genes were enriched in plant hormone signal transduction, the plant mitogen-activated protein kinase (MAPK) signal pathway, carotenoid, diterpene biosynthesis and other plant hormone synthesis pathways.

### 3.8. Differential Genes Related to Reactive Oxygen Species in MAPK Plant Signaling Pathway

As an important part of cell signal transduction, ROS are inevitably produced during the normal growth and development of plants and under stress. A certain concentration of ROS is necessary during plant growth, but if excessive ROS cannot be cleared in time, it will cause oxidative damage to cells [9]. H_2_O_2_, as a signal substance, can enter cells through aquaporins. In cells, H_2_O_2_ triggers reactive oxygen species (ROS) signals and responses, thus interfering with ROS stabilization [10]. MAPK signaling acts on receptor-like protein kinases (RLKs) and downstream of ROS signals to regulate ROS-related gene expression and programmed cell death (PCD) [43]. ROS signaling is closely related to the MAPK cascade pathway. H_2_O_2_ can induce and activate some MAPKs, and the MAPK cascade pathway can also regulate the dynamic balance of ROS [44]. In tobacco, the two signaling pathways, NPK1–MEK1–Ntf6 and Mek2–SIPK, can promote the accumulation of ROS by inducing the expression of the *NbRbohb* gene, thus enhancing its stress resistance [45]. In *Arabidopsis*, *AtMPK8* is also involved in regulating the dynamic balance of ROS, and the MKK1–MPK6 signaling pathway is involved in inducing *CAT1* expression and H_2_O_2_ production in the ABA signaling pathway [46,47].

**Figure 7:**
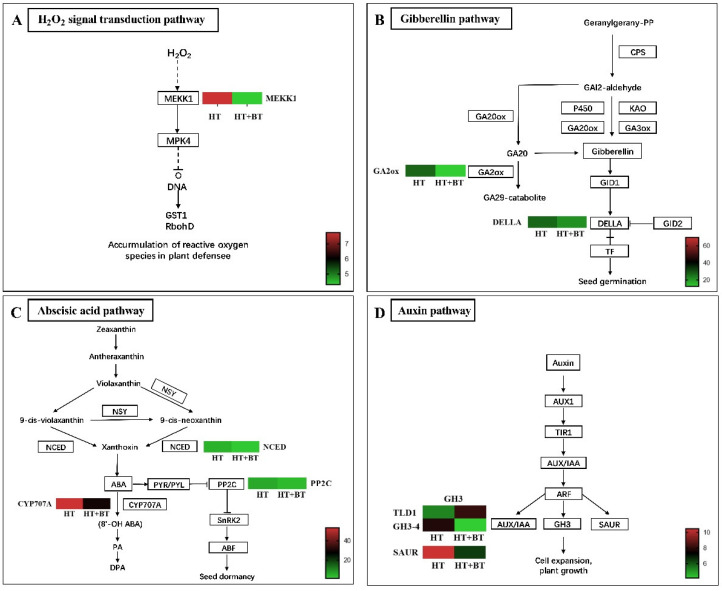
Expression profiles of DEGs in reactive oxygen species accumulation and three endogenous hormone-related pathways: (**A**) expression profiles of DEGs related to the H_2_O_2_ signal transduction pathway under HT and HT + BT treatments; (**B**) expression profiles of DEGs related to the GA pathway under HT and HT + BT treatments; (**C**) expression profiles of DEGs related to the ABA pathway under HT and HT + BT treatments; (**D**) expression profiles of DEGs related to the IAA pathway under HT and HT + BT treatments. The sample names are shown at the bottom of the figure. We indicate changes in expression levels by changes in color: green indicates a lower expression level, whereas red indicates a higher expression level. All data shown reflect the average mean of three biological replicates (*n* = 3). Means with different letters in each treatment represent a significant difference of *p* ≤ 0.05.

Heat stress produces an oxidative stress response in seeds, which leads to the production of large amounts of reactive oxygen species, which ultimately inhibit seed germination. Therefore, reducing the accumulation of excess reactive oxygen species due to heat stress is an effective way to improve seed germination rates under stress. Our previous results show that 10 mM of betaine soaking significantly increased the activity of some antioxidant enzymes in seeds under heat stress and reduced the MDA content to promote rice seed germination. In addition to the enzymatic aspects, according to our transcriptomic results (Figure 7A), betaine infiltration significantly downregulated the expression level of *MEKK1* in the H_2_O_2_ signaling pathway in the plant MAPK signaling pathway. It further reduces the production of reactive oxygen species under heat stress and improves seed adaptation to high temperatures.

### 3.9. Differential Genes Related to Hormone Signaling Pathway

To further explore the action pathway between betaine and plant hormones, we analyzed the differentially expressed genes related to plant hormones. Among the differential genes related to GAs, one is expressed in the GA synthesis pathway, and two are expressed in the GA signal transduction pathway (Figure 7B).

According to the gene expression heat map, betaine infiltration under heat stress significantly downregulated the expression level of gibberellin (*Gibberellin 2-beta-dioxygenase* (*OsGA2ox3*)) [48] and inhibited GA inactivation. We verified this by the significantly higher GA content in the betaine treatment compared with the heat-stress treatment. The betaine treatment decreased the expression level of *DELLA* [49] in the GA signal pathway, and it enhanced the output of the GA signal. In the ABA synthesis pathway, betaine induced the decrease in the expression level of *9-cis-epoxycarotenoid dioxygenase* (*NCED*) [50], the rate-limiting enzyme of ABA synthesis, and inhibited the synthesis of ABA (Figure 7C). However, at the same time, betaine also induced the downregulation of the *(+)—abscisic acid 8′–hydroxylase* (*CYP707A*) gene [51] in the ABA metabolic pathway, slowing down the metabolic rate of ABA.

In addition, in the ABA signal transduction pathway, the expression levels of three genes of *protein phosphatase 2c* (*PP2C*) [52] were downregulated in the betaine treatment, and the ABA output signal was weakened. In the IAA signal transduction pathway, we observed the expressions of two IAA auxin early-response genes (*GH3*) [53], in which the expression level of *TLD1* [54] in the HT treatment was significantly lower than that in the HT+BT treatment, while *GH3–4* [53] was significantly higher than that in the HT+BT treatment (Figure 7D). Moreover, the expression level of *SAUR* [55] was significantly higher in the HT+BT treatment than in the HT treatment. The high expression of *TLD1* promotes IAA inactivation and reduces the level of IAA in seeds. These results are consistent with the assay results of the phytohormone levels.

### 3.10. Validation of DEGs by qRT-PCR

**Figure 8:**
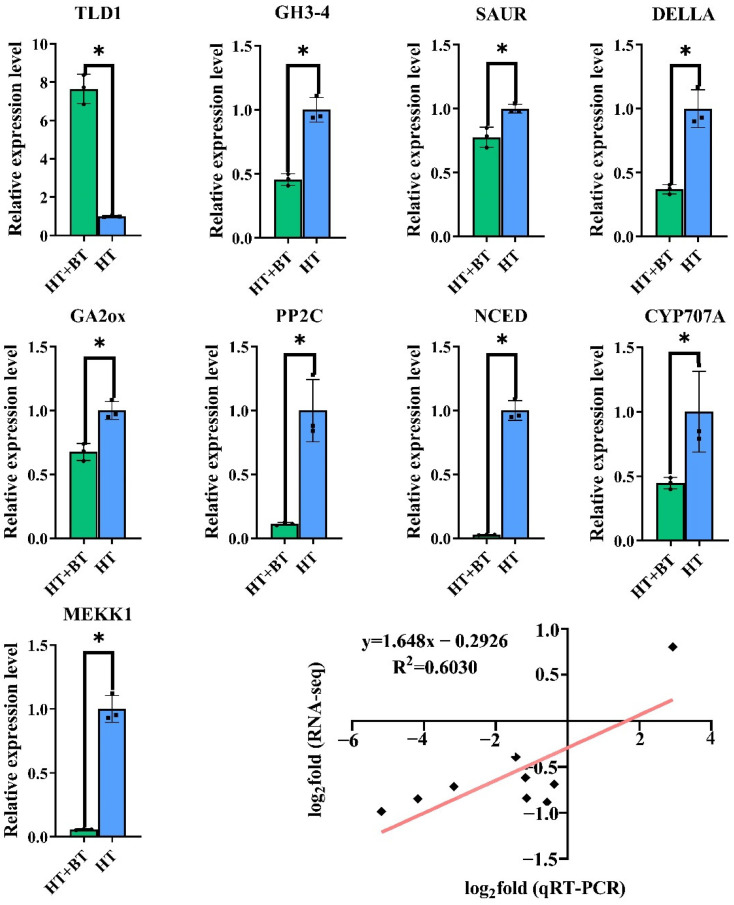
The expressions of 9 differentially expressed genes in the H_2_O_2_ pathway and 3 endogenous hormone pathways were randomly verified by qRT-PCR. The x-axis is the name of the treatment, in which HT is the 38 °C non-soaking treatment, and HT + BT is the 38 °C betaine seed-soaking treatment. The y-axis shows the relative expression of a specific gene relative to the reference gene actin. The last panel shows the results of the RNA-seq\qRT-PCR correlation analysis (lower right corner). The x-axis is the log_2_-fold-change value of the qRT-PCR, and the y-axis is the log_2_-fold-change value of the RNA-seq. According to the Pearson correlation (*R*^2^ = 0.60; *p* < 0.05), there is a significant positive correlation between the multiple changes in expression. Each parameter * indicates significant statistical difference, * (*p* < 0.05).

To verify the accuracy and reproducibility of the RNA-seq results, we randomly selected nine genes in the pathways related to seed germination for qRT-PCR validation (Figure 8). We calculated the expression levels of the selected genes using the 2^−ΔΔCt^ method. According to the qRT-PCR results, the expression levels of nine DEGs were consistent with the results of the RNA-seq, confirming the reproducibility of the data.

## 4. Discussion

### 4.1. Seed Germination

Seed germination is an important stage in the life cycle of higher plants and is one of the periods with the greatest sensitivity to the external environment. Under the condition of sufficient water, seeds will break dormancy and start germination after sensing the appropriate ambient temperature. High temperatures above 35 °C will inhibit the germination of rice seeds. The germination performance of 304 lettuce materials cultured at 21 °C, 28 °C and 35 °C for 40 h were compared. The results showed that the seeds of all 304 materials germinated at 21 °C, with an average germination rate of 87.72%. The average germination rate decreased to 42.84% at 28 °C and 1.01% at 35 °C [56]. The germination percentage of *B. subalternans* weed seeds reached above 77% for a wide alternating temperature (15/20 °C to 30/35 °C night/day). The highest germination and uniformity occurred at 25/30 °C night/day. Only 11% of the seeds germinated at a temperature of 35/40 °C night/day [57].

According to our seed germination test, when the external ambient temperature was 35 °C or below, the germination of rice seeds was not greatly affected, and the germination rate of all the treated seeds could be maintained at more than 90%. In a previous study, a drying temperature of 35°C was safe for rice seeds with high initial moisture content, whereas higher drying temperatures (41 °C and 47 °C) remarkably reduced rice seed vigor. The metabolism of ROS, antioxidant enzymes, GA, ABA and α-amylase may be closely involved in the regulation of drying temperature on the seed vigor of rice seeds with high initial moisture content [58]. However, when the temperature reached 38 °C, the germination of most rice seeds was significantly inhibited, and there was almost no germination. Under heat stress, plants accumulate some soluble substances to adapt to the stress, and betaine is one of the quaternary amines that is effective at alleviating heat stress [23]. Because rice cannot accumulate betaine naturally, we investigated the effect of betaine on rice seed germination under heat stress by exogenous seed immersion. We found the best effect at a concentration of 10 mM, which demonstrated that the betaine immersion efficiently increased the rice seed germination under heat stress. We further investigated the regulatory mechanism of the betaine promotion of seed germination under heat stress by transcriptome analysis. Compared with the control treatment, the 10 mM betaine-soaked seeds significantly induced the differential expressions of 528 genes under heat stress, of which 332 genes were downregulated, and 196 genes were upregulated. The betaine regulated the seed germination mainly by downregulating related genes. According to the GO function analysis of the differential genes, the 10 mM betaine soaking treatment under heat stress mainly affected the cellular and metabolic processes in the biological processes; the cells, membranes, membrane parts and organelles in the cell components; binding in the molecular function; and the expressions of genes related to catalytic activity to promote seed germination.

### 4.2. Physiological Changes of Rice under Heat Stress

Heat stress will cause plants to produce a heat stress response, which disturbs the structural and metabolic integrity of cells to damage cell homeostasis and then causes irreversible damage to plants [59]. At the physiological level, heat stress leads to the excessive production of ROS in mitochondria and chloroplasts through disordered electron transmission, which induces lipid peroxidation to cause serious damage to the cell membrane and eventually causes cell death [60]. Therefore, enhancing the ability to eliminate excess ROS caused by heat stress is an effective defense measure to alleviate heat stress.

Soaking seeds in 10 mM of betaine considerably reduced the MDA content in rice seeds and the level of membrane lipid peroxidation under 38 °C heat stress. The activities of SOD, POD and CAT in the antioxidant enzyme system were significantly increased, which enhanced the ability to remove excess ROS. According to the results of this study′s KEGG enrichment analysis, soaking rice seeds in betaine reduced the expression of *MEKK1* in the H_2_O_2_ signaling pathway and prevented the buildup of reactive oxygen species. On the one hand, a total of 10 mM of betaine can enhance the ability of scavenging reactive oxygen species by increasing the activity of antioxidant enzymes under heat stress. A previous study had confirmed that 10 mM exogenous betaine could effectively improve the germination potential and germination rate of *medicago sativa* seeds under drought stress, which were significantly increased by 46.8% and 30.5% compared with the control [61]. Some researchers also found that 10 mM exogenous betaine did not significantly promote the germination rate of *elymus nutans* seeds under 100 mM NaCl stress but could significantly increase the shoot length of seeds [62]. Previous research also confirmed that betaine, either applied exogenously or accumulated in vivo in *codA*-transgenic seeds, enhanced the expression of heat-shock genes and improved tolerance of high temperatures in tomato seeds during germination [22]. On the other hand, it inhibits the accumulation of reactive oxygen species by downregulating the expression of *MEKK1*. In addition, heat stress leads to a decline in the osmotic adjustment ability of crops. 

Soluble protein is an important small molecular substance that participates in osmotic adjustment when crops are stressed and that participates in the relevant physiological repair after rehydration [63]. After soaking seeds with 10 mM of betaine, the content of soluble protein in the rice seeds substantially increased, and the osmotic adjustment ability was enhanced. Physiologically speaking, the 10 mM betaine treatment increased the activity of antioxidant enzymes and decreased the level of MDA, improving the removal of excess reactive oxygen species, thereby reducing the degree of membrane lipid peroxidation, and increased the content of soluble protein, enhancing osmotic regulation and promoting rice seed germination under heat stress.

### 4.3. Synthesis and Metabolism of IAA, GAs and ABA under Heat Stress

The germination of rice seeds is regulated by plant hormones. ABA is a typical plant defense hormone that can improve the stress resistance of plants by inducing resistance and that can also regulate seed dormancy and grain maturation [64]. GAs positively promote seed germination, and they regulate seed germination with ABA in an antagonistic manner. IAA was recently found to be the second hormone that positively regulates seed dormancy after ABA, and it also responds to the induction of heat stress. The hormone level in cells is regulated by the balance between biosynthesis and catabolism. According to the KEGG enrichment analysis, betaine soaking induced the differential expressions of some genes in carotenoid and diterpene biosynthesis and plant hormone signal transduction pathways under heat stress, which were related to ABA synthesis and metabolism, GA metabolism and IAA metabolism. *NCED* is a key rate-limiting enzyme in the ABA synthesis pathway. *NCED* catalyzes the cleavage of C40 9–cis–epoxy carotenoids, 90 CIS neoflavin and 9–cis–violet, which are considered to be the main regulatory steps of ABA biosynthesis [50]. Under heat stress, the ABA levels in seeds soaked with 10 mM betaine were significantly lower than those in the control. According to the transcriptome results, the betaine treatment significantly reduced the expression level of the *NCED* gene and inhibited the synthesis of ABA. ABA is metabolically inactivated by hydroxylation or coupling. The hydroxylation of 8′–methyl is the main step in the catabolic pathway of ABA, which is catalyzed by *CYP707A*, a member of cytochrome *P450* monooxygenase [51]. The betaine treatment not only downregulated the expression level of the *NCED* gene, but also inhibited the expression of *CYP707A*. The inhibition of *NCED* gene expression reduces the ABA level and then promotes seed germination. At the same time, a decrease in the *CYP707A* gene expression level means that the ABA metabolism has slowed down, which ensures that rice seeds still maintain a certain ABA level under heat stress to trigger the defense mechanism that requires ABA stimulation to reduce heat-stress damage. Betaine substantially increased the GA levels under heat stress. The *OsGA2ox3* gene belongs to a small polygenic family encoding GAs 2–oxidase, which is a major catabolic enzyme in plants and can convert active GAs into inactive GAs [65]. The betaine treatment substantially decreased the expression level of the *OsGA2ox3* gene and inhibited the inactivation of GAs. We did not find the expressions of differential genes in the synthesis pathway of GAs, which indicates that betaine mainly increases the level of GAs by inhibiting the inactivation of GAs, rather than by promoting their biosynthesis. 

In response to the induction of heat stress, the IAA levels increased, while the betaine treatment substantially reduced them. In general, the IAA levels in plant cells are regulated in three different ways: dynamic balance, polar transport and auxin response [66]. In this experiment, there were no differential gene expressions in the biosynthesis and polar transport pathways of IAA, but we found that *GH3* family genes were differentially expressed in the IAA signal transduction pathway. *GH3–4* is very sensitive to changes in the IAA level. After the increase in the IAA level, the expression level of *GH3–4* is one of the most substantial [53]. *TLD1* encodes an IAA amination synthase. In the *TLD1–D* mutant, the expression of *TLD1* was significantly increased, which promoted the chelation of IAA with amino acids and reduced the content of free IAA [54]. The decrease in the IAA level in the betaine treatment under heat stress may be related to the high expression of *TLD1*, which suggests that betaine may regulate the level of IAA in plant cells through the auxin response. In terms of the regulation of seed germination by plant hormones, betaine reduces the level of ABA and increases the level of GAs by inhibiting the synthesis of ABA and the inactivation of GAs under heat stress, promotes the inactivation of IAA to reduce the level of IAA, and finally, promotes the germination of rice seeds.

### 4.4. Expression and Crosstalk of IAA, GA and ABA Signals

Plant hormones transmit endogenous and environmental signals through specific signal pathways to trigger output responses [67]. According to the KEGG enrichment analysis, the betaine induced the expressions of differential genes in the IAA, GA and ABA signal transduction pathways under heat stress. The expressions of these differential genes affect hormone signal transduction. In the IAA signal transduction pathway, the *GH3* expression was significantly upregulated in the betaine treatment, while the *SAUR* expression was significantly downregulated. The decrease in the IAA level is related to the upregulation of *TLD1* expression in the *GH3* gene family. *SAUR*, as an auxin early-response gene, participates in the regulation of cell elongation and growth. The overexpression of *SAUR* can promote the elongation of *Arabidopsis* cells [55]. *SAUR* promotes cell elongation and is related to *PP2C* in the ABA signal transduction pathway. *SAUR* inhibits the activity of *PP2C* and leads to the elevated activation of AHA2 phosphorylation, thereby derepressing PM H^+^–ATPases (e.g., AHA2) to acidify the cell wall and ultimately promote elongation growth [52]. In this study, we found that the expression level of *SAUR* was downregulated in response to decreased growth hormone levels; however, at the same time, the expression level of *PP2C* was also downregulated by decreased ABA levels. The promotion of seed germination by betaine under heat stress indirectly indicates the promotion of elongated cell growth, which may be mainly caused by the downregulation of the *PP2C* expression levels. 

**Figure 9:**
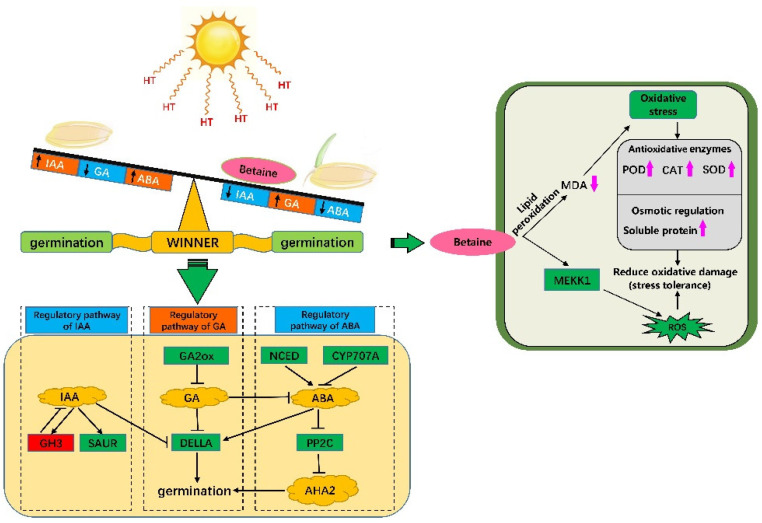
Regulatory pathways of 10 mM exogenous betaine enhance the protrusion vigor of rice seeds under heat stress.

Under heat stress, the expression level of *DELLA* in the GA signal transduction pathway was downregulated in the betaine treatment. DELLA protein is an inhibitor of GA signal transduction. In the presence of GAs, the GAs bind to the *GA–INSENSITIVE DWARF 1* (*GID1*) receptor and cause conformational changes that trigger *DELLA* degradation or that lead to its inactivation, ultimately allowing the output of the GA signaling pathway [49]. Notably, ABA can inhibit the expression of GA signaling by increasing the stability of *DELLA*, thereby inhibiting the growth of *Arabidopsis* roots [68]. In addition, the GA stimulation of *Arabidopsis* root elongation requires the involvement of growth hormone. GA-induced root elongation was inhibited when the stem tip, which is the main source of growth hormone, was removed; however, this inhibition was reversed when growth hormone was reapplied. The participation of auxin in GA-induced root elongation is completed by promoting the degradation and inactivation of DELLA protein, which is a prerequisite for GAs to stimulate root elongation [69]. This suggests that betaine can promote the degradation of DELLA protein under heat stress and enhance the output of the GA signal through three aspects: first, by increasing the level of GAs, promoting the combination of GAs and *GID1* to degrade or inactivate DELLA protein; second, by inhibiting the promoting effect of ABA on the stability of DELLA protein; third, through the DELLA protein degradation pathway participated in by IAA. According to our results, the crosstalk of plant hormones also occurs in the seed germination stage under stress, and it can coordinate the balance between signals through exogenous substances to adapt to changes in the external environment. This provides a feasible solution to alleviate the plant-growth crisis caused by global warming. The regulatory pathways of 10 mM exogenous betaine enhance the protrusion vigor of rice seeds under heat stress, as shown in Figure 9. In the future, we will explore the molecular regulatory mechanisms of betaine with respect to rice seed heat tolerance. 

## 5. Conclusions

In conclusion, according to our study, under 38 °C heat stress, the 10 mM betaine soaking treatment could alleviate the heat stress and promote the germination of rice seeds. In terms of physiology, the 10 mM betaine seed-soaking treatment promoted rice seed germination by increasing the SOD, POD and CAT antioxidant enzyme activities, decreasing the MDA content, increasing the soluble protein content to reduce ROS accumulation and mitigate membrane lipid peroxidation, and enhancing the osmoregulatory capacity to alleviate heat stress. By comparing the transcriptomes of the seed coleoptile and mesocotyl elongation stage, we obtained many DEGs involved in seed germination. According to the differential expression analysis, the betaine downregulated key genes in the H_2_O_2_ signaling pathway to reduce the accumulation of reactive oxygen species to mitigate the oxidative damage caused by heat stress. In addition, the betaine treatment affected the expressions of key genes related to the synthesis, metabolism and signal transduction of three endogenous hormones (GAs, IAA and ABA), increased the level of GAs, decreased the levels of IAA and ABA, and enhanced the output of the GA signal in rice seeds through the signaling crosstalk among the three hormones, which ultimately promoted the germination of rice seeds. The qRT-PCR results and the detection of the endogenous hormone levels further validated the expression patterns of these key genes. Our study reveals the molecular mechanism of betaine in regulating rice seed germination under heat stress, which provides an effective and feasible way to ensure the normal production of rice under the global warming trend.

## Figures and Tables

**Table 1:** Primers for qRT-PCR expression analysis.

Gene Name	Gene ID	Forward Primer	Reverse Primer
*Os11g32520*	AP014967	TCGTCCCCGTCATGAACAAG	GAAGTGGCGGCTCTTGTAGT
*Os05g42150*	AK101932	CCACCTACTTCAGCCCCAAG	GCCAAGCTATCACAGGTCGT
*Os06g45950*	XM_015786598	ATGAAGCGTCTCCTCAGGC	GAGGGAGGAGGAGGAGGAC
*Os01g62460*	XM_015763808	ATGGTCATGGATGCTGGGGT	CCGTCGCGAACCCATTGG
*Os01g55240*	XM_015793860	CGGGTTCTTCAAGGTCGTCA	ATGTCGCCATTGAACCCGAT
*Os05g46040*	XM_015784016	CATGTGGTGGTTGCCAACTG	TTCAATCCTTGCCCGCTCAT
*Os03g44380*	XM_015776052	GTGGTGCTCGACAAGGAGAA	CAGAGGTGGAAGCAGAAGCA
*Os02g47470*	AB277270	CCTCGCAACCAAGTACAGGT	ACTCCTGCTCGGTGTTCTTG
*Os03g49630*	XM_015774845	AGAAAGCGATGCCCTCCAAA	CCCTCGGCATGCACTATCAT

**Table 2:** Output and quality of sample sequencing data.

Sample	Total Raw Reads (Mil.)	Total Clean Reads (Mil.)	Total Clean Bases (Gb)	Clean Reads Q20 (%)	Clean Reads Q30 (%)	Clean Reads Ratio (%)
HT1	49.08	45.37	6.81	96.97	89.08	92.45
HT2	49.08	45.2	6.78	97.14	89.56	92.10
HT3	49.08	45.05	6.76	97.08	89.46	91.80
HT + BT1	47.33	44.39	6.66	97.08	89.14	93.79
HT + BT2	44.19	41.11	6.17	97.11	89.40	93.03
HT + BT3	47.33	43.94	6.59	97.16	89.48	92.84

## Data Availability

All of the data is contained within the article.
